# Treatment of Some Pleural Adhesions

**Published:** 1918-12

**Authors:** 


***The Treatment of some Pleural Adhesions in the Course of Intrathoracic Interventions.*** By R. Le Fort. *Bulletin de la Société de Chirurgie de Paris.* June 18, 1918.

The author reports the following case :

A soldier wounded in the chest by a shell-fragment suffered from purulent pleurisy. After this was healed, he was forwarded to Dr. Le Fort for the removal of the missile.

X-ray control detected the projectile in the neighborhood of the hilum, together with an intrathoracic drain. After opening the chest, a pleural symphysis was discovered; this was easily overcome, but the drain could not be found in the large pleural cavity. On searching it, a kind of tumor with thick walls was discovered in the external and posterior part of the lung, protruding into the pleural cavity. A drain 13 cm. long was removed from this tumor. After this removal, the patient suffered from a mediastinal emphysema extending from the lids and wrists to the scrotum. He healed perfectly well some weeks after.

The author had never met with foreign bodies enclosed in the tissues and protruding into the free large pleural cavity, with no inflammatory proceedings or fistula. The cavity including the drain in the present case proved to have exactly the shape and size of the pleural cavity.

This is an example of pleural adhesions limiting an infected and septic zone around some foreign body. Adhesions of this sort are useful and act essentially as a means of protection for the rest of the chest. The author insists, therefore, on the fact that pleural adhesions must be operated upon, in some cases, but sometimes also must be left undamaged. In some cases, pleural adhesions need to be freed to obtain a wider space for some operation. Adhesions intruding between the lungs and the ribs or diaphragm might also better be operated upon, as they may hinder the expansion of the lungs while breathing. But they must be left untouched when they are limited to the inferior part of the lung, as they act in those cases to keep the lung from retracting around the hilum. Just so, the operating of a total pleural symphysis extending all around the lung should never be practised, for after such an operation the lung would generally collapse entirely, and all efforts might often prove unable to bring its expansion. In such a case the surgeon should always keep at least a certain zone of adherences both at the top and bottom of the lungs, so as to maintain the normal vertical size of these and hinder their collapsing.

Adhesions must be considered in certain cases as a true defence of the organism against some infective process, and be spared, therefore, whenever this seems advisable and possible.

